# Bis{bis­(azido-κ*N*)bis­[bis­(pyridin-2-yl-κ*N*)amine]cobalt(III)} sulfate dihydrate

**DOI:** 10.1107/S2056989016003662

**Published:** 2016-03-08

**Authors:** Fatima Setifi, Jacqueline M. Knaust, Zouaoui Setifi, Rachid Touzani

**Affiliations:** a Laboratoire de Chimie, Ingénierie Moléculaire et Nanostructures (LCIMN), Université Ferhat Abbas Sétif 1, Sétif 19000, Algeria; b Department of Chemistry, Mathematics and Physics, Clarion University, 840 Wood Street, Clarion, PA 16214, USA; c Laboratoire de Chimie Appliquée et Environnement, LCAE-URAC18, COSTE, Faculté des Sciences, Université Mohamed Premier, BP524, 60000, Oujda, Morocco, and, Faculté Pluridisciplinaire Nador BP 300, Selouane, 62702, Nador, Morocco

**Keywords:** crystal structure, coordination compound, Co^III^ complex, di-2-pyridyl­amine (dpa), azide, hydrogen bonding, supra­molecular structure

## Abstract

The search for new mol­ecular materials with inter­esting magnetic properties, using the pseudohalide azide ion and di-2-pyridyl­amine (dpa, C_10_H_9_N_3_) as a chelating ligand, led to the synthesis and structure determination of the title compound, [Co(N_3_)_2_(dpa)_2_]_2_SO_4_·2H_2_O. The crystal structure comprises discrete [Co(dpa)_2_(N_3_)_2_]^+^ cations, sulfate anions, as well as H_2_O solvent mol­ecules. The Co^III^ cations display a slightly distorted octa­hedral coordination sphere defined by two N atoms from azide anions and four N atoms from the pyridyl rings of two dpa ligands. In the crystal, extensive C—H⋯O, N—H⋯O, and O—H⋯O inter­actions result in supra­molecular sheets that lie parallel to the *ab* plane. The sheets are further linked through O—H⋯N inter­actions between the water mol­ecules of one sheet and azide anions of another sheet, forming a supra­molecular framework.

## Abstract 2

The crystal structure of bis­{bis­(azido-κ*N*)bis­[bis­(pyridin-2-yl-κ*N*)amine]­cobalt(III)} sulfate dihydrate is comprised of discrete [Co(dpa)_2_(N_3_)_2_]^+^ cations, SO_4_^2−^ anions and solvent water mol­ecules in a 2:1:2 ratio; extensive hydrogen-bonding inter­actions link the species into a three-dimensional supra­molecular framework.

## Chemical context   

In recent years, mol­ecular magnetism has attracted great attention due to the inter­est in designing new mol­ecular materials with inter­esting magnetic properties and potential applications (Kahn, 1993 ▸; Miller & Gatteschi, 2011 ▸). Connecting paramagnetic ions by use of bridging polynitrile or pseudohalide ligands is an important strategy in the design of such materials (Setifi *et al.*, 2002 ▸, 2003 ▸, 2013 ▸, 2014 ▸; Miyazaki *et al.*, 2003 ▸; Benmansour *et al.*, 2008 ▸, 2009 ▸; Yuste *et al.*, 2009 ▸). As a short bridging ligand and efficient superexchange mediator, the pseudohalide azide ion has proven to be very versatile and diverse in both coordination chemistry and magnetism. It can link metal ions in μ-1,1 (end-on, EO), μ-1,3 (end-to-end, EE) and μ-1,1,1 coordination modes among others, and effectively mediate either ferromagnetic or anti­ferromagnetic coupling. Many azide-bridged systems with different dimensionality and topology have been synthesized by using various auxiliary ligands, and a great diversity of magnetic behaviors have been demonstrated (Ribas *et al.*, 1999 ▸; Gao *et al.*, 2004 ▸; Liu *et al.*, 2007 ▸; Mautner *et al.*, 2010 ▸). In view of the possible roles of the versatile azido ligand, we have been inter­ested in using it in combination with other chelating or bridging neutral co-ligands to explore their structural and electronic characteristics in the field of mol­ecular materials exhibiting inter­esting magnetic exchange coupling. During the course of attempts to prepare such complexes with di-2-pyridyl­amine, we isolated the title compound, whose structure is described herein.
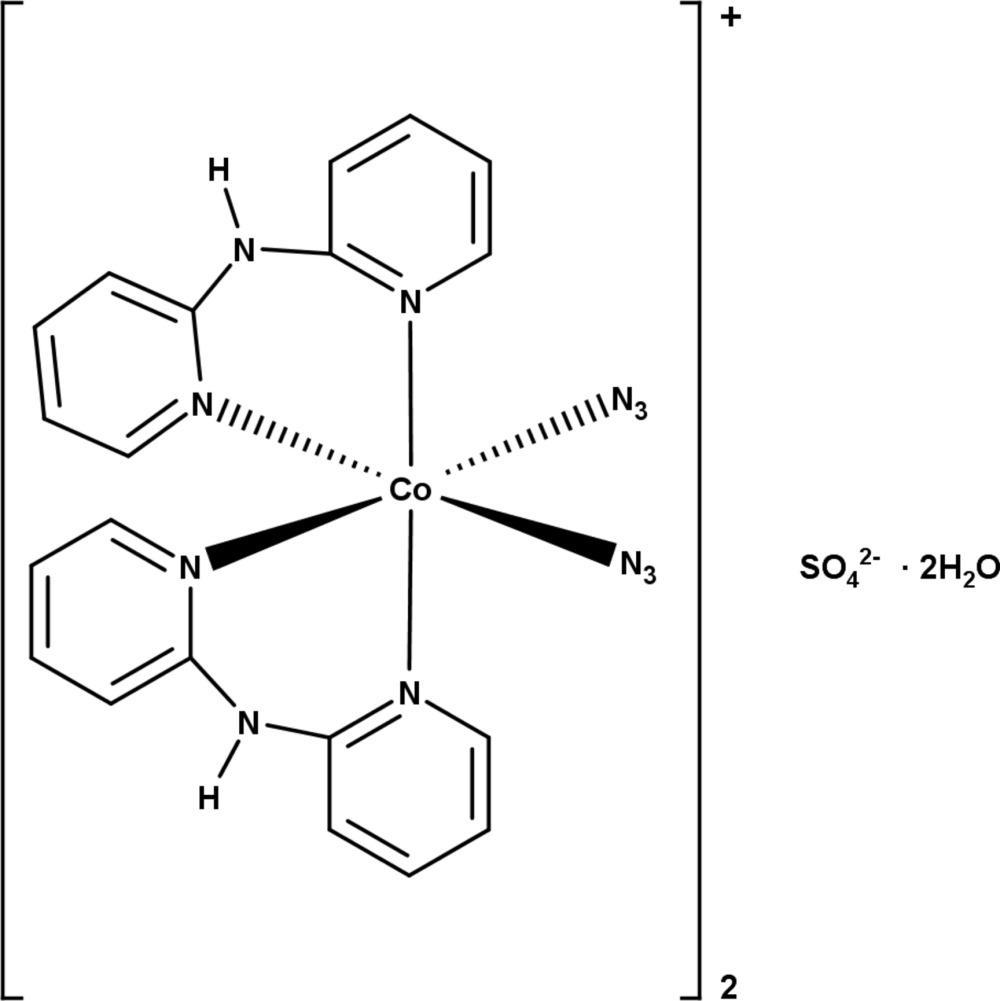


## Structural commentary   

**Figure 1:**
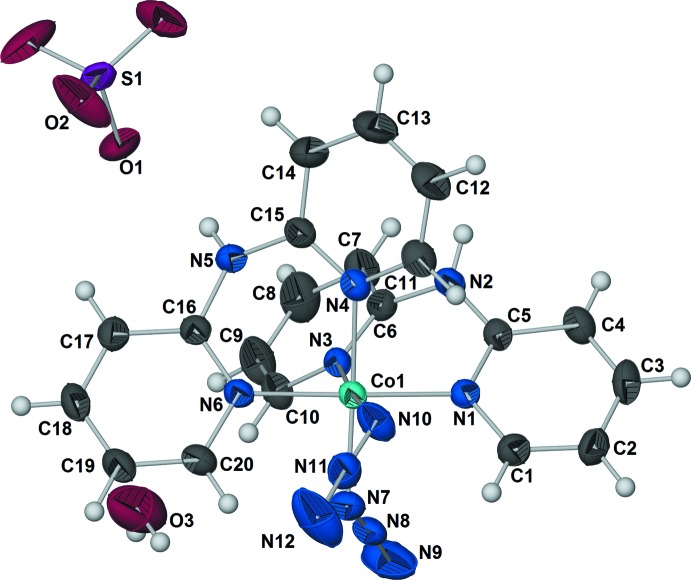
The mol­ecular entities in the crystal structure of the title compound drawn with displacement ellipsoids at the 50% probability level for non-H atoms and spheres of arbitrary size for H atoms. [Symmetry code: (iv) −*x* + 1, *y*, −*z* + 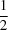.]

The structure of the title compound is composed of discrete [Co(dpa)_2_(N_3_)_2_]^+^ cations, SO_4_^2−^ anions, and solvent water mol­ecules in a 2:1:2 ratio (Fig. 1 ▸). The sulfate anion is located on a twofold rotational axis, and all other atoms lie on general positions. The central Co^III^ ion has an approximately octa­hedral coordination geometry formed by four N-donors from the pyridyl rings of two chelating bidentate dpa ligands and two N-donors from the terminal azide anions with the *cisoid* angles ranging from 84.97 (8) to 94.15 (8)° and *transoid* angles ranging from 174.02 (8) to 176.36 (8)° (Table 1 ▸). While the bite angles of the dpa ligands are both less than 90° the smallest *cisoid* angle observed is for N4—Co1—N10 (Table 1 ▸), and the pyridyl ring containing N4 and azide anion containing N10 are involved in a weak C—H⋯N inter­action (C11—H11⋯N10) (Table 2 ▸). The two pyridyl rings of each chelating dpa ligand coordinate to the metal in a *cis*-disposition, and the azide anions are also coordinating *cis* to each other.

A similar arrangement of ligands is observed in four of the five transition metal compounds reported with coordination environments comprised of two chelating dpa ligands and two terminal azide anions [CSD refcodes: ATAFEG (Du *et al.*, 2004 ▸); EYOWEU (Villanueva *et al.*, 2004 ▸); HUFNUR (Du *et al.*, 2001 ▸); JANPOE (Bose *et al.*, 2005 ▸); ATAFEG01 and EYOWEU01 (Rahaman *et al.*, 2005 ▸)]; in the fifth compound, [Cu(dpa)_2_(N_3_)_2_]·2H_2_O, the two pyridyl rings of each chelating dpa ligand still coordinate to the metal in a *cis*-disposition, but the azide anions are coordinated *trans* to each other [CSD refcode: XUYWIX (Du *et al.*, 2003 ▸)]. The six Co—N bond lengths are comparable to those observed in [Co(dpa)_2_(N_3_)_2_]ClO_4_ [CSD refcode; HUFNUR; Du *et al.*, 2001 ▸)] and range from 1.9334 (18) to 1.9699 (17) Å with a mean bond length of 1.955 Å (Table 1 ▸). Both of the coordin­ating azide anions are nearly linear with N—N—N bond angles of 175.7 (2) and 175.3 (3)° for N7—N8—N9 and N10—N11—N12, respectively (Table 1 ▸). Not unexpectedly, the Co1—N7—N8 and Co1—N10—N11 bond angles are 122.05 (15) and 126.09 (17)°, respectively, and the N—N bond lengths are slightly longer for the bonds involving nitro­gen atoms coord­inating to the Co^III^ ion at 1.208 (3) and 1.181 (2) Å for N7—N8 and N10—N11, respectively, *versus* 1.142 (3) and 1.148 (3) Å for N8—N9 and N11—N12, respectively (Dori & Ziolo, 1973 ▸) (Table 1 ▸).

**Figure 2:**
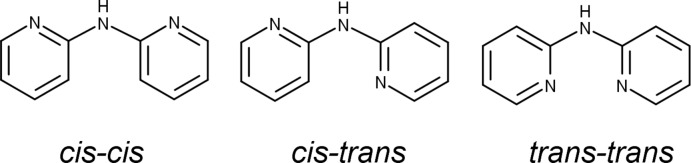
Conformations of dpa. *Cis* and *trans* refer to the relation of the pyridyl N atoms to the amine N atom.

**Figure 3:**
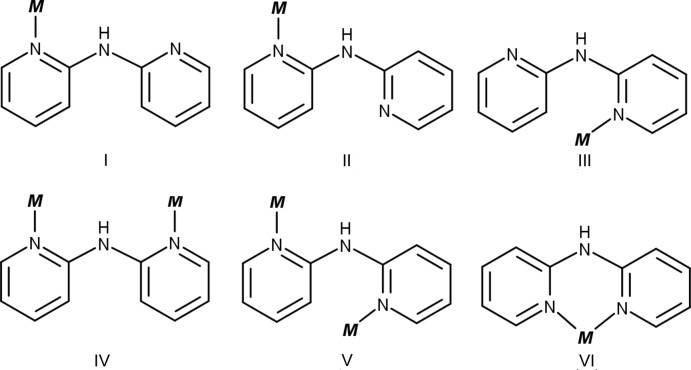
Possible coordination modes of dpa involving only pyridyl N atoms. Only modes **I**–**II** and **IV**–**VI** are observed with transition metals.

Three conformations are known for dpa, *cis–cis*, *cis–trans*, or *trans–trans* (Fig. 2 ▸); *cis* and *trans* refer to the relation of the pyridyl nitro­gen atoms to the amine nitro­gen (Gornitzka & Stalke, 1998 ▸). Several bonding modes are possible involving just the pyridyl nitro­gen atoms (Fig. 3 ▸). Only bonding modes **I**–**II** and **IV–VI** are observed for dpa with transition metals, but additional bonding modes are possible for anionic dpa involving coordination *via* the amide nitro­gen, and there are also a few reports of coordination at the deprotonated *ortho* carbon of one of the pyridyl rings (Brogden & Berry, 2016 ▸). In the title compound, as in the vast majority of structures where neutral dpa coordinates to a transition metal (see *Database Survey*), dpa adopts the *trans–trans* conformation and acts as a chelating ligand in bonding mode **VI**. The flexible nature of the dpa ligand is well recognized (Carranza *et al.*, 2008 ▸; Du *et al.*, 2004 ▸; Wang *et al.*, 2009 ▸), and as is often observed for coordinating dpa ligands, each dpa ligand in the title complex is quite distorted from planarity due to folding of the pyridyl rings about the line connecting the amino nitro­gen atom and the metal cation with a 46.18 (5)° angle between plane 1 (defined by atoms Co1/N1/C1–C5/N2) and plane 2 (defined by atoms N2/C6–C10/N3/Co1) and a 37.40 (6)° angle between plane 3 (defined by atoms Co1/N4/C11–C15/N5) and plane 4 (defined by atoms N5/C16–C20/N6/Co1) (Table 3 ▸). For dpa ligands coordinating to a metal atom *via* bonding mode **VI**, a wide range of pyridine centroid–amine nitro­gen–pyridine centroid (Py_cent_—N_a_—Py_cent_) angles and pyridine nitro­gen–metal–pyridine nitro­gen (N_py_—*M*—N_py_) bite angles are reported, but no simple trend between the two angles is observed (Brogden & Berry, 2016 ▸).

In [Co(dpa)_2_(N_3_)_2_]^+^, the Py1_cent_—N_2_—Py3_cent_ and N1—Co1—N3 angles are 120.92 (7) and 86.48 (7)°, and the Py4_cent_—N5—Py5_cent_ and N4—Co1—N6 angles are 125.49 (7) and 88.08 (7)° (Py1, Py3, Py4, and Py6 are the pyridyl rings containing N1, N3, N4, and N6 respectively) (Table 1 ▸). The C—N—C angles around the amino nitro­gen are larger than expected for a trigonal planar nitro­gen atom at 123.44 (17)° for C5—N2—C6 and 125.87 (19)° for C15—N5—C16, but N—C—N angles at the ring junctions are closer to 120° [N1—C5—N2 = 119.38 (18)°; N2—C6—N3 = 119.1 (2)°; N4—C15—N5 = 119.30 (18)°; N5—C16—N6 = 120.16 (18)°], and the metal lies less than 2.5° from the lone-pair direction for each pyridyl nitro­gen atom [C5—N1—Co1 = 120.94 (14); C6—N3—Co1 = 120.49 (14); C15—N4—Co1=122.47 (15); C16—N6—Co1 = 121.35 (13)°; Table 1 ▸]. Both of the dpa ligands form six-membered chelate rings with boat conformations. For chelate ring –Co1–N1–C5–N2–C6–N3–, atoms Co1 and N2 lie 0.868 (3) and 0.336 (3) Å below the mean plane defined by atoms N1/C5/C6/N3, and for chelate ring –Co–N4–C15–N5–C16–N6–, atoms Co1 and N5 lie 0.770 (3) and 0.278 (3) Å above the mean plane defined by atoms N4/C15/C16/N6 (Table 3 ▸).

## Supra­molecular features   

**Figure 4:**
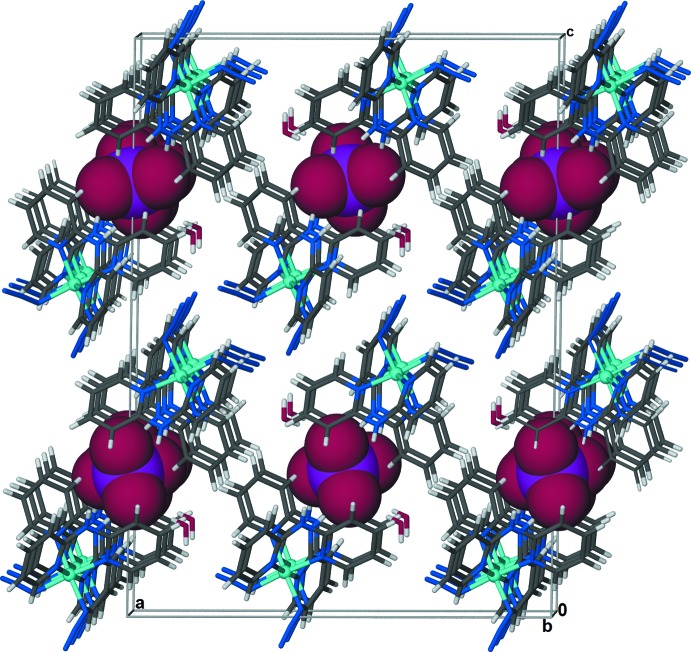
The sulfate anions, highlighted in space-filling mode, are sandwiched between two symmetry related layers of complex cations and water mol­ecules.

**Figure 5:**
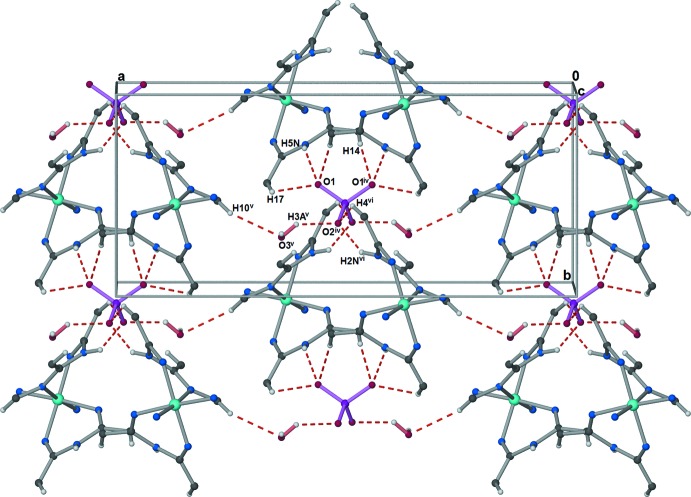
Extensive C—H⋯O, N—H⋯O, and O—H⋯O hydrogen bonding, represented by dashed red lines, links the anions, complex cations, and water mol­ecules into sheets parallel to the *ab* plane. For clarity, the pyridine C and H atoms not involved in hydrogen-bonding inter­actions have been omitted, and only the N atom coordinating to the Co^III^ cation is shown for the azide anions. [Symmetry codes: (iv) −*x* + 1, *y*, −*z* + 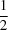; (v) −*x* + 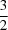, *y* + 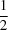, −*z* + 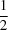; (vi) −*x* + 1, *y* + 1, −*z* + 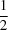.]

**Figure 6:**
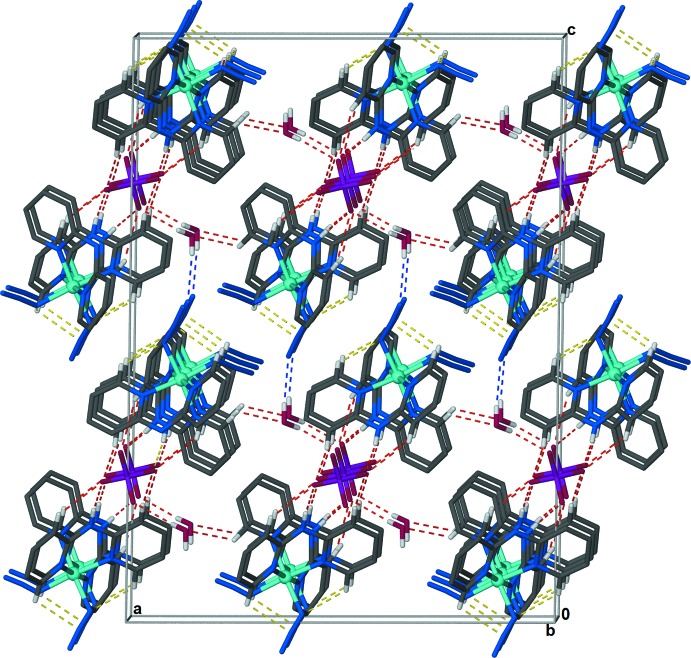
The two-dimensional supra­molecular sheets that lie parallel to the *ab* plane are linked *via* O—H⋯N inter­actions, represented by dashed blue lines, between the water mol­ecules of one sheet and azide anions of another sheet to form a three-dimensional supra­molecular framework. The discussed C—H⋯N hydrogen bonds between neighboring dpa ligands and azide anions within the coordination sphere of the Co^III^ cation are represented by dashed yellow lines, and the C—H⋯O, N—H⋯O, and O—H⋯O hydrogen bonds linking the anions, complex cations, and water mol­ecules into sheets are represented by dashed red lines. For clarity hydrogen atoms not involved in hydrogen-bonding inter­actions have been omitted.

Stabilizing C—H⋯N inter­actions (C11—H11⋯N10, C20—H20⋯N7, C20—H20⋯N11) are observed between neighboring dpa ligands and azide anions within the coordination sphere of the Co^III^ cation (Table 2 ▸). The complex cations and water mol­ecules aggregate into layers parallel to the *ab* plane, and each [Co(C_10_H_9_H_3_)_2_(N_3_)_2_]^+^ complex cation inter­acts with one water mol­ecule through a C—H⋯O hydrogen bond (C10—H10⋯O3). The sulfate anions are sandwiched between two symmetry-related layers of complex cations and water mol­ecules (Fig. 4 ▸). Each sulfate anion inter­acts with two water mol­ecules and four [Co(C_10_H_9_H_3_)_2_(N_3_)_2_]^+^ cations through twelve hydrogen bonds (Fig. 5 ▸). As the sulfate anion is located on a twofold rotational axis, only six of the twelve hydrogen bonds are unique (O3—H3*A*⋯O2^ii^, N2—H2*N*⋯O2^iii^, N5—H5*N*⋯O1, C4—H4⋯O2^iii^, C14—H14⋯O1^iv^, and C17—H17⋯O1). The extensive C—H⋯O, N—H⋯O, and O—H⋯O inter­actions result in two-dimensional supra­molecular sheets parallel to the *ab* plane (Fig. 5 ▸). Finally, the sheets are linked *via* O—H⋯N inter­actions between the water mol­ecules of one sheet and the azide anions of another sheet (O3—H3*B*⋯N12^i^), forming a supra­molecular framework (Fig. 6 ▸).

## Database survey   

Free dpa crystallizes as one of several polymorphs, but only in the *cis–trans* conformation with an intra­molecular C—H⋯N hydrogen bond between the two pyridyl rings [CSD refcodes: DPYRAM (Johnson & Jacobson, 1973 ▸); DPYRAM01 (Pyrka & Pinkerton, 1992 ▸); DPYRAM03 and DPYRAM04 (Schödel *et al.*, 1996 ▸)]. Theoretical calculations by Wu *et al.* (2013 ▸) give the *cis–trans* conformation at 2.5 and 8.0. kcal mol^−1^ more stable than the *cis–cis* and *trans–trans* conformations, respectively, and the authors suggest that the instability of free dpa in the *trans–trans* conformation is due to repulsive inter­actions between of the pyridyl nitro­gen lone pairs. However, when dpa coordinates to a transition metal, the *trans–trans* conformation is preferred. A survey of the Cambridge Structural Database (CSD; Groom & Allen, 2014 ▸) returned 735 hits for structures involving a dpa ligand coordinating to a transition metal cation *via* at least one of its pyridyl rings (structures involving anionic dpa and coordination to a metal *via* the amide nitro­gen were excluded from the search). Of the 735 hits, only 15 structures involve dpa acting as a monodentate ligand in either the *cis–cis* or *cis–trans* conformations (bonding modes **I** and **II**, respectively) are reported. Dpa acts as a bridging ligand in only three structures in either the *cis–cis* or *cis–trans* conformations (bonding modes **IV** and **V**, respectively). No structures are observed with dpa in bonding mode **III**. In the remainder of the structures, dpa adopts the *trans–trans* conformation and acts as a chelating ligand in bonding mode **VI**.

**Figure 7:**
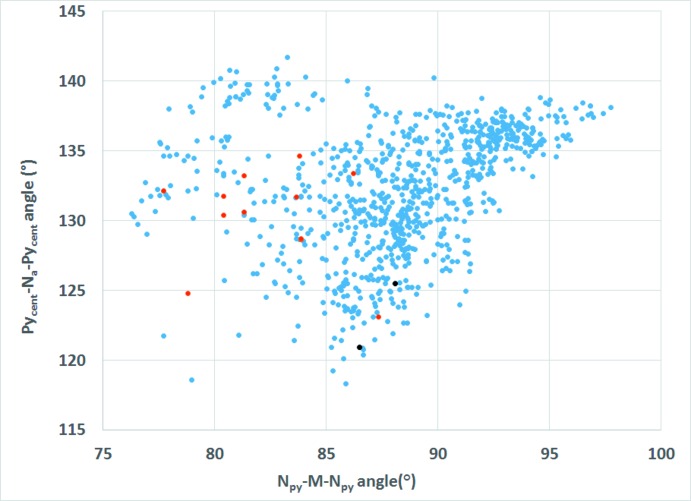
Scatter plot of py_cent_—N_a_—py_cent_ angles *versus* N_py_—*M*—N_py_ bite angles for all transition metal complexes reported to the CSD with dpa in coordination mode **VI**. Blue dots represent all complexes with dpa coordinating in bonding mode **VI** to a transition metal. Red dots represent compounds where the metal has a coordination environment similar to the title compound: two dpa in bonding mode **VI** and two terminal azide anions. Black dots represent the title compound.

**Figure 8:**
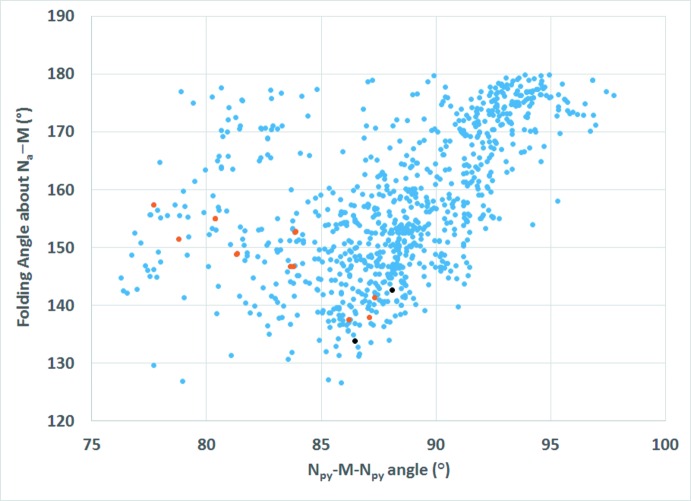
Scatter plot of the folding angles about N_a_—*M versus* N_py_—*M*—N_py_ bite angles for all transition metal complexes reported to the CSD with dpa in coordination mode **VI**. Blue dots represent all complexes with dpa coordinating in bonding mode **VI** to a transition metal. Red dots represent compounds where the metal has a coordination environment similar to the title compound: two dpa in bonding mode **VI** and two terminal azide anions. Black dots represent the title compound.

**Figure 9:**
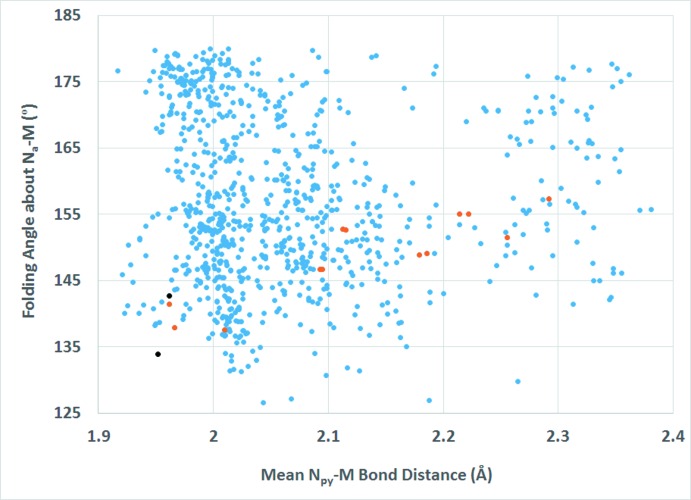
Scatter plot of the folding angles about N_a_—*M versus* mean *M*—N_py_ bond length for all transition metal complexes reported to the CSD with dpa in coordination mode **VI**. Blue dots represent all complexes with dpa coordinating in bonding mode **VI** to a transition metal. Red dots represent compounds where the metal has a coordination environment similar to the title compound: two dpa in bonding mode **VI** and two terminal azide anions. Black dots represent the title compound.

As mentioned in the *Structural commentary*, dpa is a flexible ligand and adopts a wide range of Py_cent_—N_a_—Py_cent_ and N_py_—*M*—N_py_ bite angles in transition metal complexes. A comparison of these angles in the title compound to those observed in all structures reported to the CSD involving dpa coordinating to a transition metal in bonding mode **VI** reveals no simple trend (Brogden & Berry, 2016 ▸) (Fig. 7 ▸). Comparison of the folding angle about N_a_—*M* versus the N_py_—*M*—N_py_ bite angle (Fig. 8 ▸) as well as the folding angle about N_a_—*M versus* the mean N_py_—M distance (Fig. 9 ▸) in the title compound to those observed in all structures reported to the CSD involving dpa coordinating to a transition metal in bonding mode **VI** also supports the flexible nature of dpa as a chelating ligand; however, no simple trend between the folding angle and the bite angle or the folding angle and the mean N_py_—*M* distance is indicated.

A more narrow search for structures involving at least one terminal azide anion and one dpa ligand in bonding mode **VI** within the coordination sphere of a transition metal cation returned 30 hits for 25 unique structures. Of the 25 structures, 23 involve *M* ^II^ cations; there is one report for Co^III^ [CSD refcode: HUFNUR (Du *et al.*, 2001 ▸)] and another report for Pt^IV^[CSD refcode: YATYOJ (Ha, 2012 ▸)]. Five structures are reported where the metal cation has a coordination sphere similar to that of the title compound. In each case, an approximately octa­hedral coordination geometry is formed by four N-donors from the pyridyl rings of two dpa ligands and two N-donors from terminal azide anions. In [*M*(dpa)_2_(N_3_)_2_]·H_2_O with *M* = Mn [CSD refcode: JANPOE (Bose *et al.*, 2005 ▸)], Ni [CSD refcodes: EYOWEU (Villanueva *et al.*, 2004 ▸) and EYOWEU01 (Rahaman *et al.* 2005 ▸)], and Zn [CSD refcodes: ATAFEG (Du *et al.*, 2004 ▸) and ATAFEG01 (Rahaman *et al.*, 2005 ▸)], neutral complexes are observed. In each case, the azide anions coordinate to the metal cation in a *cis*-fashion, and hydrogen bonding, face-to-face π–π stacking, and edge-to-face C–H⋯π inter­actions result in a three-dimensional supra­molecular framework. In [Cu(dpa)_2_(N_3_)_2_]·2H_2_O, the azide anions coordinate to the Cu^II^ ion weakly in a *trans*-fashion, resulting in a tetra­gonally elongated octa­hedral coordination sphere for the Cu^II^ ion, and hydrogen bonding and face-to-face π–π stacking inter­actions result in two-dimensional supra­molecular sheets that lie parallel to the *bc*-plane [CSD refcode: XUYWIX (Du *et al.*, 2003 ▸)]. [Co(dpa)_2_(N_3_)_2_]ClO_4_ is most closely related to the title complex in that the Co^III^ ions are coordinated by two chelating dpa ligands and two azide anions in a *cis*-fashion to form [Co(dpa)_2_(N_3_)_2_]^+^ complex cations [CSD refcode: HUFNUR (Du *et al.*, 2001 ▸)]. The structure is stabilized by strong N—H⋯O inter­actions between the complex cation and perchlorate anions. Consideration of additional weak C—H⋯N inter­actions between the cations (which were not discussed by the authors) results in supra­molecular ribbons that run parallel to the *c* axis.

## Synthesis and crystallization   

The title compound was synthesized hydro­thermally under autogenous pressure from a mixture of cobalt(II) sulfate hepta­hydrate (28 mg, 0.1 mmol), di-2-pyridyl­amine (17 mg, 0.1 mmol) and sodium azide NaN_3_ (13 mg, 0.2 mmol) in water–methanol (4:1 *v*/*v*, 20 ml). The mixture was sealed in a Teflon-lined autoclave and heated at 423 K for two days and cooled to room temperature at 10 K h^−1^. The crystals were obtained in *ca* 20% yield based on cobalt.

***CAUTION!*** Although not encountered in our experiments, azido compounds of metal ions are potentially explosive. Only a small amount of the materials should be prepared, and it should be handled with care.

## Refinement   

Crystal data, data collection and structure refinement details are summarized in Table 4 ▸. All aromatic H atoms were positioned geometrically and refined using a riding model with C—H = 0.93 Å and *U*_iso_(H) = 1.2*U*_eq_(C). The N—H and O—H-atoms were located in difference Fourier maps and then refined as riding on the carrying nitro­gen or oxygen atom with *U*_iso_(H) = 1.2*U*_eq_(N) or *U*_iso_(H) = 1.5*U*_eq_(O). Two reflections considered to be affected by beam stop inter­ference, 0 0 2 and 2 0 0, were omitted from the refinement.

## Supplementary Material

Crystal structure: contains datablock(s) I. DOI: 10.1107/S2056989016003662/zl2656sup1.cif

Structure factors: contains datablock(s) I. DOI: 10.1107/S2056989016003662/zl2656Isup2.hkl

CCDC reference: 1457112

Additional supporting information:  crystallographic information; 3D view; checkCIF report

## Figures and Tables

**Table 1:** Selected geometric parameters (Å, °)

Co1—N1	1.9534 (17)	Co1—N10	1.951 (2)
Co1—N3	1.9680 (18)	N7—N8	1.208 (3)
Co1—N4	1.9699 (17)	N8—N9	1.142 (3)
Co1—N6	1.9533 (16)	N10—N11	1.181 (2)
Co1—N7	1.9334 (18)	N11—N12	1.148 (3)
			
N1—Co1—N3	86.48 (7)	N7—N8—N9	175.7 (2)
N1—Co1—N4	91.77 (7)	N10—N11—N12	175.3 (3)
N1—Co1—N6	176.36 (8)	Co1—N7—N8	122.05 (15)
N1—Co1—N7	90.68 (7)	Co1—N10—N11	126.09 (17)
N1—Co1—N10	89.46 (8)	C5—N2—C6	123.44 (17)
N3—Co1—N4	92.28 (7)	C15—N5—C16	125.87 (19)
N3—Co1—N6	89.89 (7)	N1—C5—N2	119.38 (18)
N3—Co1—N7	93.31 (8)	N2—C6—N3	119.1 (2)
N3—Co1—N10	175.02 (7)	N4—C15—N5	119.30 (18)
N4—Co1—N6	88.08 (7)	N5—C16—N6	120.16 (18)
N4—Co1—N7	174.02 (8)	C5—N1—Co1	120.94 (14)
N4—Co1—N10	84.97 (8)	C6—N3—Co1	120.49 (14)
N6—Co1—N7	89.82 (7)	C15—N4—Co1	122.47 (15)
N6—Co1—N10	94.15 (8)	C16—N6—Co1	121.35 (13)
N7—Co1—N10	89.61 (9)		

**Table 2:** Hydrogen-bond geometry (Å, °)

*D*—H⋯*A*	*D*—H	H⋯*A*	*D*⋯*A*	*D*—H⋯*A*
O3—H3*B*⋯N12^i^	0.86	2.24	3.095 (4)	173
O3—H3*A*⋯O2^ii^	0.86	2.02	2.832 (3)	158
N2—H2*N*⋯O2^iii^	0.86	1.94	2.708 (2)	147
N5—H5*N*⋯O1	0.86	2.08	2.742 (2)	134
C4—H4⋯O2^iii^	0.93	2.67	3.346 (3)	130
C10—H10⋯O3	0.93	2.51	3.203 (3)	131
C11—H11⋯N10	0.93	2.55	2.911 (3)	104
C14—H14⋯O1^iv^	0.93	2.49	3.399 (3)	165
C17—H17⋯O1	0.93	2.56	3.261 (3)	132
C20—H20⋯N7	0.93	2.42	2.837 (3)	107
C20—H20⋯N11	0.93	2.60	3.101 (3)	114

Symmetry codes: (i) 
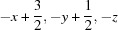
; (ii) 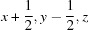; (iii) 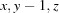; (iv) 
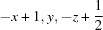
.

**Table 3:** Deviations of atoms from the least-squares planes and angle between planes (Å, °) Note: (*) an atom that was not used to define the plane.

Atom	Plane 1^*a*^	Plane 2^*b*^	Plane 3^*c*^	Plane 4^*d*^	Plane 5^*e*^	Plane 6^*f*^
Co1	−0.1338 (10)	−0.1647 (11)	0.1345 (11)	0.1699 (11)	−0.8678 (25)*	0.7703 (27)*
N1	0.0362 (16)				−0.0061 (9)	
N2	0.1351 (14)	0.1438 (13)			−0.3360 (28)*	
N3		0.0556 (14)			0.0061 (9)	
N4			−0.0521 (16)			0.0091 (10)
N5			−0.0985 (14)	−0.1311 (14)		0.2776 (29)*
N6				−0.0726 (16)		−0.0091 (9)
C1	0.0931 (18)					
C2	0.0613 (18)					
C3	−0.0736 (18)					
C4	−0.1218 (18)					
C5	0.0036 (14)				0.0067 (10)	
C6		0.0126 (18)			−0.0067 (10)	
C7		−0.1227 (20)				
C8		−0.1026 (22)				
C9		0.0577 (20)				
C10		0.1204 (18)				
C11			−0.1083 (18)			
C12			−0.0322 (20)			
C13			0.0909 (23)			
C14			0.0806 (22)			
C15			−0.0150 (20)			−0.0101 (11)
C16				−0.0248 (19)		0.0101 (11)
C17				0.1239 (18)		
C18				0.1050 (18)		
C19				−0.0558 (18)		
C20				−0.1144 (18)		
	Angle Between planes (°)					
	Plane 1	Plane 3				
Plane 2	46.18 (5)					
Plane 4		37.400 (6)				

Least-squares planes (*x*, *y*, *z* in crystal coordinates) and r.m.s. deviation of fitted atoms (*a*) 17.6944 (0.0050) *x* − 3.3356 (0.0034) *y* + 6.4703 (0.0175) *z* = 11.4816 (0.0019); 0.0936. (*b*) 10.8385 (0.0107) *x* − 6.6693 (0.0036) *y* − 9.4426 (0.0122) *z* = 5.6865 (0.0073); 0.1087. (*c*) 3.1401 (0.0101) *x* − 6.8035 (0.0036) *y* + 16.3074 (0.0138) *z* = 2.5612 (0.0063); 0.0853. (*d*) 14.1733 (0.0085) *x* − 4.0917 (0.0033) *y* + 13.8837 (0.0152) *z* = 9.4863 (0.0050): 0.1087. (*e*) 14.7476 (0.0151) *x* − 5.8441 (0.0074) *y* − 0.5348 (0.0288) *z* = 9.6107 (0.0074); 0.0064. (*f*) 9.4731 (0.0138) *x* − 6.0570 (0.0073) *y* + 14.4127 (0.0340) *z* = 5.8151 (0.0082); 0.0096.

**Table 4:** Experimental details

Crystal data	
Chemical formula	[Co(N_3_)_2_(C_10_H_9_N_3_)_2_]_2_SO_4_·2H_2_O
*M*_r_	1102.88
Crystal system, space group	Monoclinic, *C*2/*c*
Temperature (K)	293
*a*, *b*, *c* (Å)	19.9014 (4), 8.7044 (2), 27.1181 (5)
β (°)	90.753 (1)
*V* (Å^3^)	4697.25 (17)
*Z*	4
Radiation type	Mo *K*α
μ (mm^−1^)	0.83
Crystal size (mm)	0.26 × 0.17 × 0.09

Data collection	
Diffractometer	Bruker APEXII CCD
Absorption correction	Multi-scan (*SADABS*; Bruker, 2009 ▸)
*T*_min_, *T*_max_	0.808, 0.875
No. of measured, independent and observed [*I* > 2σ(*I*)] reflections	50250, 6893, 3940
*R*_int_	0.096
(sin θ/λ)_max_ (Å^−1^)	0.705

Refinement	
*R*[*F* ^2^ > 2σ(*F* ^2^)], *wR*(*F* ^2^), *S*	0.043, 0.094, 0.90
No. of reflections	6893
No. of parameters	331
H-atom treatment	H-atom parameters constrained
Δρ_max_, Δρ_min_ (e Å^−3^)	0.49, −0.52

Computer programs: *APEX2* and *SAINT* (Bruker, 2009 ▸), *SHELXS97* (Sheldrick, 2008 ▸), *SHELXL2014* (Sheldrick, 2015 ▸) and *X-SEED* (Barbour, 2001 ▸).

## supplementary crystallographic information

### Crystal data

**Table:**

[Co(N_3_)_2_(C_10_H_9_N_3_)_2_]_2_SO_4_·2H_2_O	*F*(000) = 2264
*M**_r_* = 1102.88	*D*_x_ = 1.560 Mg m^−^^3^
Monoclinic, *C*2/*c*	Mo *K*α radiation, λ = 0.71073 Å
*a* = 19.9014 (4) Å	Cell parameters from 7924 reflections
*b* = 8.7044 (2) Å	θ = 2.5–24.6°
*c* = 27.1181 (5) Å	µ = 0.83 mm^−^^1^
β = 90.753 (1)°	*T* = 293 K
*V* = 4697.25 (17) Å^3^	Block, red
*Z* = 4	0.26 × 0.17 × 0.09 mm

### Data collection

**Table:**

Bruker APEXII CCD diffractometer	6893 independent reflections
Radiation source: sealed tube	3940 reflections with *I* > 2σ(*I*)
Graphite monochromator	*R*_int_ = 0.096
φ and ω scans	θ_max_ = 30.1°, θ_min_ = 2.5°
Absorption correction: multi-scan (*SADABS*; Bruker, 2009)	*h* = −28→28
*T*_min_ = 0.808, *T*_max_ = 0.875	*k* = −12→12
50250 measured reflections	*l* = −38→38

### Refinement

**Table:**

Refinement on *F*^2^	0 restraints
Least-squares matrix: full	Hydrogen site location: mixed
*R*[*F*^2^ > 2σ(*F*^2^)] = 0.043	H-atom parameters constrained
*wR*(*F*^2^) = 0.094	*w* = 1/[σ^2^(*F*_o_^2^) + (0.0412*P*)^2^] where *P* = (*F*_o_^2^ + 2*F*_c_^2^)/3
*S* = 0.90	(Δ/σ)_max_ = 0.001
6893 reflections	Δρ_max_ = 0.49 e Å^−^^3^
331 parameters	Δρ_min_ = −0.52 e Å^−^^3^

### Special details

**Table:**

Geometry. All e.s.d.'s (except the e.s.d. in the dihedral angle between two l.s. planes) are estimated using the full covariance matrix. The cell e.s.d.'s are taken into account individually in the estimation of e.s.d.'s in distances, angles and torsion angles; correlations between e.s.d.'s in cell parameters are only used when they are defined by crystal symmetry. An approximate (isotropic) treatment of cell e.s.d.'s is used for estimating e.s.d.'s involving l.s. planes.

### Fractional atomic coordinates and isotropic or equivalent isotropic displacement parameters (Å^2^)

**Table:**

	*x*	*y*	*z*	*U*_iso_*/*U*_eq_	
Co1	0.62803 (2)	0.08164 (3)	0.07843 (2)	0.02579 (9)	
S1	0.5000	0.58900 (9)	0.2500	0.0362 (2)	
O1	0.55892 (9)	0.4950 (2)	0.24043 (8)	0.0631 (6)	
O2	0.48452 (10)	0.6853 (3)	0.20752 (7)	0.0753 (7)	
O3	0.86029 (11)	0.2512 (3)	0.15996 (8)	0.0925 (8)	
H3A	0.8961	0.2070	0.1708	0.139*	
H3B	0.8603	0.2324	0.1289	0.139*	
N1	0.60151 (9)	−0.13172 (19)	0.06724 (7)	0.0277 (4)	
N2	0.57170 (9)	−0.1581 (2)	0.15044 (7)	0.0316 (4)	
H2N	0.5366	−0.1727	0.1680	0.038*	
N3	0.66233 (8)	0.01273 (19)	0.14314 (6)	0.0263 (4)	
N4	0.53936 (9)	0.13277 (19)	0.10540 (7)	0.0282 (4)	
N5	0.58666 (9)	0.30108 (19)	0.16366 (7)	0.0323 (4)	
H5N	0.5886	0.3159	0.1950	0.039*	
N6	0.65632 (8)	0.29126 (18)	0.09387 (7)	0.0274 (4)	
N7	0.71278 (10)	0.0457 (2)	0.04634 (7)	0.0374 (5)	
N8	0.75070 (10)	−0.0551 (2)	0.05942 (8)	0.0404 (5)	
N9	0.78932 (12)	−0.1472 (3)	0.06960 (11)	0.0730 (9)	
N10	0.58775 (10)	0.1370 (2)	0.01490 (8)	0.0420 (5)	
N11	0.61222 (10)	0.2161 (2)	−0.01536 (8)	0.0369 (5)	
N12	0.63226 (14)	0.2907 (3)	−0.04682 (10)	0.0783 (9)	
C1	0.60855 (11)	−0.1984 (3)	0.02241 (9)	0.0356 (6)	
H1	0.6312	−0.1445	−0.0019	0.043*	
C2	0.58399 (12)	−0.3408 (3)	0.01124 (9)	0.0394 (6)	
H2	0.5908	−0.3845	−0.0196	0.047*	
C3	0.54857 (12)	−0.4186 (3)	0.04718 (10)	0.0418 (6)	
H3	0.5298	−0.5141	0.0402	0.050*	
C4	0.54117 (11)	−0.3552 (3)	0.09263 (9)	0.0354 (5)	
H4	0.5165	−0.4054	0.1166	0.042*	
C5	0.57136 (10)	−0.2132 (2)	0.10264 (8)	0.0276 (5)	
C6	0.62535 (11)	−0.0810 (2)	0.17145 (8)	0.0292 (5)	
C7	0.64101 (14)	−0.1050 (3)	0.22072 (9)	0.0443 (6)	
H7	0.6126	−0.1632	0.2403	0.053*	
C8	0.69828 (15)	−0.0430 (3)	0.24052 (10)	0.0560 (8)	
H8	0.7092	−0.0582	0.2736	0.067*	
C9	0.74015 (13)	0.0436 (3)	0.21047 (10)	0.0492 (7)	
H9	0.7806	0.0827	0.2226	0.059*	
C10	0.72045 (11)	0.0694 (2)	0.16294 (9)	0.0357 (5)	
H10	0.7480	0.1287	0.1430	0.043*	
C11	0.48315 (11)	0.0676 (2)	0.08557 (9)	0.0364 (5)	
H11	0.4870	0.0131	0.0562	0.044*	
C12	0.42166 (12)	0.0780 (3)	0.10643 (11)	0.0476 (7)	
H12	0.3844	0.0307	0.0920	0.057*	
C13	0.41593 (13)	0.1606 (3)	0.14953 (11)	0.0590 (8)	
H13	0.3748	0.1660	0.1653	0.071*	
C14	0.47057 (12)	0.2342 (3)	0.16911 (10)	0.0494 (7)	
H14	0.4669	0.2927	0.1976	0.059*	
C15	0.53216 (11)	0.2201 (2)	0.14548 (9)	0.0323 (5)	
C16	0.63826 (10)	0.3604 (2)	0.13612 (8)	0.0277 (5)	
C17	0.67037 (11)	0.4937 (2)	0.15335 (9)	0.0343 (5)	
H17	0.6599	0.5344	0.1840	0.041*	
C18	0.71738 (11)	0.5632 (2)	0.12447 (10)	0.0382 (6)	
H18	0.7391	0.6519	0.1352	0.046*	
C19	0.73217 (11)	0.5000 (3)	0.07916 (9)	0.0378 (6)	
H19	0.7620	0.5487	0.0582	0.045*	
C20	0.70238 (11)	0.3651 (2)	0.06560 (9)	0.0331 (5)	
H20	0.7140	0.3213	0.0356	0.040*	

### Atomic displacement parameters (Å^2^)

**Table:**

	*U*^11^	*U*^22^	*U*^33^	*U*^12^	*U*^13^	*U*^23^
Co1	0.02576 (15)	0.02659 (15)	0.02507 (17)	−0.00383 (12)	0.00259 (12)	0.00118 (13)
S1	0.0488 (5)	0.0334 (4)	0.0268 (5)	0.000	0.0147 (4)	0.000
O1	0.0534 (12)	0.0569 (11)	0.0795 (16)	0.0011 (9)	0.0266 (11)	−0.0322 (11)
O2	0.0638 (13)	0.1057 (17)	0.0562 (14)	−0.0388 (12)	−0.0109 (11)	0.0498 (12)
O3	0.0682 (15)	0.146 (2)	0.0629 (16)	−0.0251 (15)	0.0010 (13)	0.0020 (15)
N1	0.0285 (9)	0.0291 (9)	0.0254 (11)	−0.0028 (8)	0.0012 (8)	−0.0009 (8)
N2	0.0323 (10)	0.0350 (10)	0.0276 (11)	−0.0087 (8)	0.0047 (8)	0.0016 (8)
N3	0.0266 (9)	0.0248 (9)	0.0276 (11)	−0.0034 (7)	0.0005 (8)	−0.0003 (8)
N4	0.0270 (10)	0.0284 (9)	0.0291 (11)	−0.0031 (8)	−0.0007 (8)	0.0026 (8)
N5	0.0343 (10)	0.0355 (10)	0.0272 (11)	−0.0066 (8)	0.0077 (9)	−0.0044 (8)
N6	0.0267 (9)	0.0274 (9)	0.0281 (11)	−0.0018 (7)	0.0037 (8)	0.0028 (8)
N7	0.0351 (11)	0.0390 (11)	0.0384 (13)	−0.0021 (9)	0.0121 (10)	−0.0002 (9)
N8	0.0339 (11)	0.0355 (11)	0.0522 (14)	−0.0056 (10)	0.0186 (10)	−0.0002 (10)
N9	0.0521 (15)	0.0533 (14)	0.114 (2)	0.0157 (13)	0.0311 (16)	0.0267 (15)
N10	0.0423 (12)	0.0517 (12)	0.0319 (12)	−0.0151 (10)	−0.0032 (10)	0.0126 (10)
N11	0.0403 (11)	0.0410 (11)	0.0293 (12)	−0.0046 (9)	−0.0040 (10)	−0.0013 (10)
N12	0.0860 (19)	0.102 (2)	0.0463 (16)	−0.0462 (17)	−0.0125 (14)	0.0355 (15)
C1	0.0356 (13)	0.0394 (13)	0.0319 (14)	−0.0042 (10)	0.0025 (11)	−0.0033 (11)
C2	0.0410 (14)	0.0396 (13)	0.0376 (15)	−0.0036 (11)	−0.0032 (12)	−0.0133 (11)
C3	0.0437 (14)	0.0332 (12)	0.0483 (17)	−0.0081 (11)	−0.0124 (12)	−0.0067 (12)
C4	0.0349 (13)	0.0337 (12)	0.0376 (15)	−0.0071 (10)	−0.0034 (11)	0.0039 (11)
C5	0.0264 (11)	0.0271 (11)	0.0291 (13)	−0.0016 (9)	−0.0019 (10)	0.0010 (9)
C6	0.0325 (12)	0.0269 (10)	0.0283 (13)	−0.0008 (10)	−0.0003 (10)	−0.0008 (10)
C7	0.0569 (16)	0.0452 (14)	0.0308 (14)	−0.0159 (12)	−0.0038 (12)	0.0051 (11)
C8	0.073 (2)	0.0597 (17)	0.0351 (16)	−0.0154 (15)	−0.0165 (15)	0.0079 (13)
C9	0.0467 (15)	0.0543 (16)	0.0461 (18)	−0.0139 (13)	−0.0202 (13)	0.0050 (13)
C10	0.0324 (12)	0.0359 (12)	0.0386 (15)	−0.0059 (10)	−0.0054 (11)	0.0029 (11)
C11	0.0330 (12)	0.0379 (13)	0.0383 (15)	−0.0012 (11)	−0.0049 (11)	0.0044 (11)
C12	0.0267 (12)	0.0571 (16)	0.0588 (19)	−0.0071 (12)	−0.0020 (12)	0.0002 (14)
C13	0.0330 (15)	0.0762 (19)	0.068 (2)	−0.0085 (14)	0.0181 (15)	−0.0124 (17)
C14	0.0375 (14)	0.0573 (16)	0.0538 (18)	−0.0063 (13)	0.0162 (13)	−0.0148 (14)
C15	0.0298 (12)	0.0297 (11)	0.0376 (14)	−0.0027 (10)	0.0052 (11)	0.0022 (10)
C16	0.0252 (11)	0.0265 (10)	0.0313 (13)	0.0023 (9)	0.0007 (10)	0.0020 (10)
C17	0.0331 (12)	0.0321 (12)	0.0376 (15)	−0.0013 (10)	−0.0011 (11)	−0.0044 (11)
C18	0.0304 (12)	0.0318 (12)	0.0522 (17)	−0.0077 (10)	−0.0040 (11)	−0.0017 (11)
C19	0.0333 (13)	0.0350 (13)	0.0452 (16)	−0.0067 (11)	0.0048 (12)	0.0056 (12)
C20	0.0291 (12)	0.0364 (12)	0.0339 (14)	−0.0034 (10)	0.0046 (10)	0.0037 (10)

### Geometric parameters (Å, º)

**Table:**

Co1—N1	1.9534 (17)	C1—H1	0.9300
Co1—N3	1.9680 (18)	C2—C3	1.387 (3)
Co1—N4	1.9699 (17)	C2—H2	0.9300
Co1—N6	1.9533 (16)	C3—C4	1.360 (3)
Co1—N7	1.9334 (18)	C3—H3	0.9300
Co1—N10	1.951 (2)	C4—C5	1.399 (3)
S1—O2	1.4544 (19)	C4—H4	0.9300
S1—O2^i^	1.4544 (19)	C6—C7	1.384 (3)
S1—O1^i^	1.4559 (17)	C7—C8	1.365 (4)
S1—O1	1.4559 (17)	C7—H7	0.9300
O3—H3A	0.8578	C8—C9	1.394 (4)
O3—H3B	0.8578	C8—H8	0.9300
N1—C5	1.342 (3)	C9—C10	1.361 (3)
N1—C1	1.356 (3)	C9—H9	0.9300
N2—C6	1.378 (3)	C10—H10	0.9300
N2—C5	1.382 (3)	C11—C12	1.358 (3)
N2—H2N	0.8600	C11—H11	0.9300
N3—C6	1.346 (3)	C12—C13	1.378 (4)
N3—C10	1.361 (3)	C12—H12	0.9300
N4—C15	1.336 (3)	C13—C14	1.364 (4)
N4—C11	1.359 (3)	C13—H13	0.9300
N5—C16	1.378 (2)	C14—C15	1.396 (3)
N5—C15	1.379 (3)	C14—H14	0.9300
N5—H5N	0.8600	C16—C17	1.402 (3)
N6—C16	1.347 (3)	C17—C18	1.369 (3)
N6—C20	1.364 (3)	C17—H17	0.9300
N7—N8	1.208 (3)	C18—C19	1.382 (3)
N8—N9	1.142 (3)	C18—H18	0.9300
N10—N11	1.181 (2)	C19—C20	1.364 (3)
N11—N12	1.148 (3)	C19—H19	0.9300
C1—C2	1.365 (3)	C20—H20	0.9300
			
N1—Co1—N3	86.48 (7)	C1—C2—H2	121.0
N1—Co1—N4	91.77 (7)	C3—C2—H2	121.0
N1—Co1—N6	176.36 (8)	C4—C3—C2	120.1 (2)
N1—Co1—N7	90.68 (7)	C4—C3—H3	120.0
N1—Co1—N10	89.46 (8)	C2—C3—H3	120.0
N3—Co1—N4	92.28 (7)	C3—C4—C5	118.9 (2)
N3—Co1—N6	89.89 (7)	C3—C4—H4	120.6
N3—Co1—N7	93.31 (8)	C5—C4—H4	120.6
N3—Co1—N10	175.02 (7)	N1—C5—C4	121.6 (2)
N4—Co1—N6	88.08 (7)	N2—C5—C4	119.04 (19)
N4—Co1—N7	174.02 (8)	N3—C6—C7	121.6 (2)
N4—Co1—N10	84.97 (8)	N2—C6—C7	119.27 (19)
N6—Co1—N7	89.82 (7)	C8—C7—C6	119.7 (2)
N6—Co1—N10	94.15 (8)	C8—C7—H7	120.1
N7—Co1—N10	89.61 (9)	C6—C7—H7	120.1
N7—N8—N9	175.7 (2)	C7—C8—C9	119.1 (3)
N10—N11—N12	175.3 (3)	C7—C8—H8	120.4
Co1—N7—N8	122.05 (15)	C9—C8—H8	120.4
Co1—N10—N11	126.09 (17)	C10—C9—C8	118.4 (2)
C5—N2—C6	123.44 (17)	C10—C9—H9	120.8
C15—N5—C16	125.87 (19)	C8—C9—H9	120.8
N1—C5—N2	119.38 (18)	C9—C10—N3	123.1 (2)
N2—C6—N3	119.1 (2)	C9—C10—H10	118.4
N4—C15—N5	119.30 (18)	N3—C10—H10	118.4
N5—C16—N6	120.16 (18)	C12—C11—N4	123.3 (2)
C5—N1—Co1	120.94 (14)	C12—C11—H11	118.4
C6—N3—Co1	120.49 (14)	N4—C11—H11	118.4
C15—N4—Co1	122.47 (15)	C11—C12—C13	118.2 (2)
C16—N6—Co1	121.35 (13)	C11—C12—H12	120.9
O2—S1—O2^i^	109.6 (2)	C13—C12—H12	120.9
O2—S1—O1^i^	107.60 (11)	C14—C13—C12	120.1 (2)
O2^i^—S1—O1^i^	110.20 (12)	C14—C13—H13	120.0
O2—S1—O1	110.20 (12)	C12—C13—H13	120.0
O2^i^—S1—O1	107.60 (11)	C13—C14—C15	118.7 (2)
O1^i^—S1—O1	111.63 (15)	C13—C14—H14	120.7
H3A—O3—H3B	103.8	C15—C14—H14	120.7
C5—N1—C1	117.87 (18)	N4—C15—C14	121.9 (2)
C1—N1—Co1	121.02 (14)	N5—C15—C14	118.8 (2)
C6—N2—H2N	118.3	N6—C16—C17	121.85 (19)
C5—N2—H2N	118.3	N5—C16—C17	117.99 (19)
C6—N3—C10	117.6 (2)	C18—C17—C16	119.2 (2)
C10—N3—Co1	121.60 (14)	C18—C17—H17	120.4
C15—N4—C11	117.58 (18)	C16—C17—H17	120.4
C11—N4—Co1	119.71 (15)	C17—C18—C19	119.2 (2)
C16—N5—H5N	117.1	C17—C18—H18	120.4
C15—N5—H5N	117.1	C19—C18—H18	120.4
C16—N6—C20	117.15 (18)	C20—C19—C18	119.1 (2)
C20—N6—Co1	120.92 (15)	C20—C19—H19	120.5
N1—C1—C2	123.1 (2)	C18—C19—H19	120.5
N1—C1—H1	118.4	C19—C20—N6	123.2 (2)
C2—C1—H1	118.4	C19—C20—H20	118.4
C1—C2—C3	118.1 (2)	N6—C20—H20	118.4

Symmetry code: (i) −*x*+1, *y*, −*z*+1/2.

### Hydrogen-bond geometry (Å, º)

**Table:**

*D*—H···*A*	*D*—H	H···*A*	*D*···*A*	*D*—H···*A*
O3—H3*B*···N12^ii^	0.86	2.24	3.095 (4)	173
O3—H3*A*···O2^iii^	0.86	2.02	2.832 (3)	158
N2—H2*N*···O2^iv^	0.86	1.94	2.708 (2)	147
N5—H5*N*···O1	0.86	2.08	2.742 (2)	134
C4—H4···O2^iv^	0.93	2.67	3.346 (3)	130
C10—H10···O3	0.93	2.51	3.203 (3)	131
C11—H11···N10	0.93	2.55	2.911 (3)	104
C14—H14···O1^i^	0.93	2.49	3.399 (3)	165
C17—H17···O1	0.93	2.56	3.261 (3)	132
C20—H20···N7	0.93	2.42	2.837 (3)	107
C20—H20···N11	0.93	2.60	3.101 (3)	114

Symmetry codes: (i) −*x*+1, *y*, −*z*+1/2; (ii) −*x*+3/2, −*y*+1/2, −*z*; (iii) *x*+1/2, *y*−1/2, *z*; (iv) *x*, *y*−1, *z*.
